# Social Closure and the Evolution of Cooperation via Indirect Reciprocity

**DOI:** 10.1038/s41598-018-29290-0

**Published:** 2018-07-24

**Authors:** Simone Righi, Károly Takács

**Affiliations:** 1 Department of Computer Science, University College London, London, WC1E 6EA United Kingdom; 2 Hungarian Academy of Sciences, MTA TK “Lendület” Research Center for Educational and Network Studies (RECENS), Budapest, 1097 Hungary

**Keywords:** Social evolution, Evolutionary theory

## Abstract

Direct and indirect reciprocity are good candidates to explain the fundamental problem of evolution of cooperation. We explore the conditions under which different types of reciprocity gain dominance and their performances in sustaining cooperation in the PD played on simple networks. We confirm that direct reciprocity gains dominance over indirect reciprocity strategies also in larger populations, as long as it has no memory constraints. In the absence of direct reciprocity, or when its memory is flawed, different forms of indirect reciprocity strategies are able to dominate and to support cooperation. We show that indirect reciprocity relying on social capital inherent in closed triads is the best competitor among them, outperforming indirect reciprocity that uses information from any source. Results hold in a wide range of conditions with different evolutionary update rules, extent of evolutionary pressure, initial conditions, population size, and density.

## Introduction

The explanation of the conditions leading to the evolution of cooperation between non-kin individuals is a fundamental problem for the biological and social sciences^1,2^. Reciprocity has been found as one major mechanism that consolidates human cooperation^3^. Two types of reciprocal behavior are considered, direct and indirect reciprocity^4^. Direct reciprocity describes that if an agent treats another kindly (unkindly), the latter tends to reciprocate the former with an action alike. When the likelihood of repeating the interaction is high, direct reciprocity strategies succeed in the most puzzling Prisoner’s Dilemma game (PD)^5–7^. The simplicity and fairness of the reciprocal Tit for Tat strategy ensures success in a wide range of conditions^1,8^. Some additional generosity that forgives for occasional mistakes is even more beneficial^9,10^.

The dyad-based heuristic of direct reciprocity requires however a perfect recall of the previous interaction. Thus, in a well-connected mid- or large-size population, the implementation of direct reciprocity becomes problematic. Memory constraints, the inherent possibility of misinterpreting partners’ behavior, and natural limits on the number of interaction partners, create space for the use of proxies such as indirect reciprocity^11^. In contrast to its direct counterpart, indirect reciprocity prescribes to help (or to retaliate) those, who helped (or cheated) somebody else^12^. Indirect strategies, that obtain and reciprocate information from interactions in which the given individual was *not* involved, enhance the chances of cooperation and social coordination^11,13–16^. Experiments confirmed the prevalence of indirect reciprocity in various settings^17–19^, such as in cyclical networks^12,20^, and found a high frequency of punishment acts by observers of interaction towards parties who defected previously^21–23^.

A precise conceptualization of indirect reciprocity can take various forms and multiple strategies or “norms” fit under this umbrella term. This includes “Give and you shall be given” (help B and anticipate help from C), “Pay back the community the help you received” (if you have been helped by B, help C), as well as reputation-based accounts as “I won’t scratch your back, if you won’t scratch their backs” (I will not help you if you did not help others) or “I help B, because he refused to give help to C, who did not help anyone”.

Indirect reciprocity strategies rely on reputational information about the partner. Publicly shared reputation is the basis of the evolution of cooperation via image scoring^11,24^. The objective image score of an individual is improved by cooperation and reduced by defection. A discriminator strategy, that conditions action on the (relative) image score of the partner, is able to gain dominance in the population and to establish large-scale cooperation^11,24–26^. The image scoring strategy, however, is unable to differentiate between defectors and good-hearted reciprocal players, who used defection to punish free-riders^26^. An indirect reciprocity strategy that corrects for this is “good standing”, according to which an individual does not lose reputation by failing to cooperate with individuals who lack good standing^13,27,28^. Good standing has outperformed image scoring in some studies^13^, but not in others^29^.

Image scores reflect objective summaries of past actions, but plain judgements on who can be considered as partners of good reputation will also do. Simple strategies that discriminate partners with “good” and “bad” reputation could take many variants. As direct reciprocity retaliates defection and rewards cooperation, indirect reciprocity strategies evaluate past actions or the resulting reputation of the interaction partner. A precise account of all simple indirect reciprocity norms that consider a partner as good or bad based on his or her action (cooperation or defection) and on the reputation of his or her previous opponent (good or bad) is analyzed by Ohtsuki and Iwasa^30–32^, who found that strategies that are labeled as “leading eight” can maintain cooperation via indirect reciprocity. All these strategies consider cooperation with good opponents as good and defection against them as bad, but the evaluation of action against bad individuals differs^30^. The strategy “stern-judging”, in particular, that prescribes defection against bad opponents as good and cooperation with them as bad behavior^33^ has been shown to be exceptionally successful^34,35^.

Indirect reciprocity covers multiple mechanisms also with regard to the *source of information* that is considered by the players. The network structure of indirect reciprocity is key to cooperation^36,37^. Indirect reciprocity that relies on social closure describes the strategy that rewards (retaliates) good (bad) actions of third parties in closed cycles. The simplest form of which is triadic reciprocity: I cooperate with new partner B, who cooperated with A, who cooperated against me in a previous interaction. Indirect reciprocity based on social closure is present in human cooperation in various forms such as circles of favor^38^, rings of gifts^39^, and local exchange trading systems^40^. Moreover, sociological research highlights that relational support works more efficiently in cohesive network structures^38,41,42^. Cooperation in cohesive structures with high transitive closure is well tractable, establishes high trust^43^, and results in safe returns to investment of nice behavior. Moreover, social closure offers the best possibility to employ effective punishment and deter others from free riding^44^. In this way, cohesion and closure function to maintain cooperative norms and social order; and as a consequence, communities with high transitive closure are characterized with more effective norms^45,46^. In addition to empirical observations in which social closure might be intertwined with other factors, existing experimental studies also underline the hypothesis that dense and cohesive structures support cooperation^44,47^.

We label indirect reciprocity that relies on social closure as Connected Reciprocity and contrast its performance with Unconnected Reciprocity that benefits from information also from directly unrelated third parties. The latter strategy does not rely on closed circles of interactions, and cooperates with partner B, if B cooperated in his previous interaction. In comparison to Connected Reciprocity, Unconnected Reciprocity has several advantageous features. First, it does not rely only on local and redundant information, being able to benefit from information from directly unrelated others. Second, it does not fall into cycles of defection induced by accidental mistakes or retaliations by conditional cooperators. These benefits are in line with the literature that highlights how larger returns of social capital can be gained from weak ties^48^ (despite the higher risk they are at), brokerage^49^, and structural holes^50,51^.

We analyze the evolutionary success of acquiring and utilizing reputational information from different sources in the most puzzling social dilemma game, the Prisoner’s Dilemma (PD). Differently from recent research, we do not use a simple donation game instead^35^ and do not include extortion strategies^52–59^, as we do not intend to determine the ultimate winner strategy. Instead, by contrasting indirect reciprocity that conditions behavior on information from direct social ties with indirect reciprocity that conditions behavior on information from indirectly related individuals, we contribute to the evaluation of social network based and of impersonal reputation systems.

## The Model

We consider a set of N agents *i* ∈ {1, …, *N*}. Each agent *i*(**T**_*i*_, **F**_*i*_) is characterized by a strategy type **T**_*i*_ and a fixed set of connections to a subset of the whole population **F**_*i*_ ⊂ *N*, which constrains the interaction with other players. The network of connections is a non-directed Erdös-Rényi (E-R) graph with a given density *λ*, i.e. each tie exists with a probability *λ* = *P*(*j* ∈ **F**_*i*_) ∀ *i*, *j*. Alternative network configurations have also been analyzed (results on a lattice are reported in Figs S12–S14).

**Figure 1:**
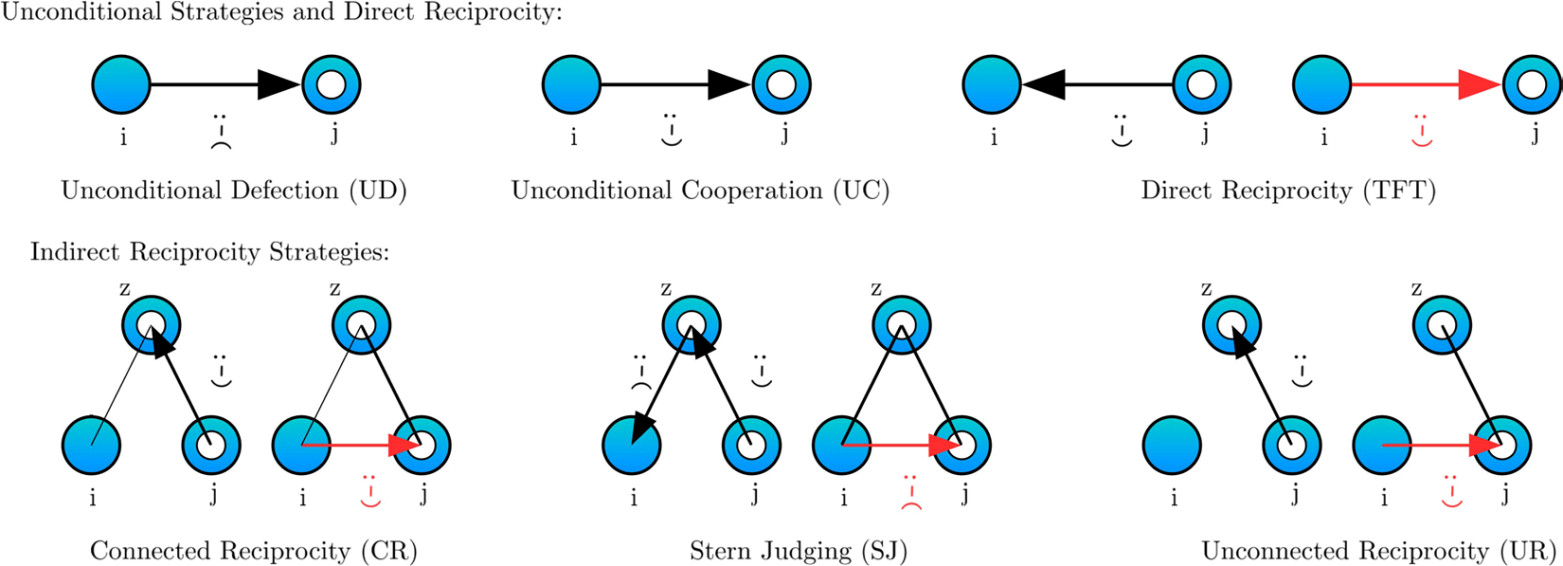
Strategies of *i*, when playing with *j*: *Unconditional Defection (UD)*: Always defects regardless of the behaviour of interaction partners. *Unconditional Cooperation (UC)*: Always cooperates regardless of the behaviour of interaction partners. *Tit for Tat (TFT)*: Reciprocates the last action of the interacting partner in the given dyadic relationship (it starts by defecting). Similar results with a generally higher cooperation rate follow if we assume that TFT starts with cooperation (Figs S1, S10 and S11). *Connected Reciprocity (CR)*: Reciprocates the last action of the interacting partner with a randomly selected common connection (i.e. one *z* ∈ **F**_*i*_ ∧ **F**_*j*_). *Unconnected Reciprocity (UR)*: Reciprocates the last action of the interacting partner with a randomly selected connection of the latter (i.e. one *z* ∈ **F**_*j*_). *Stern Judging (SJ)*: Rewards partners who cooperated with good partners with cooperation, partners who defected against good partners with defection, punishes partners who cooperated with bad partners with defection, and partners who defected with bad partners with cooperation. If there is no previous action to observe, then the indirect reciprocity strategies act randomly with 50% chance of cooperation.

Agents play a two-person Prisoner’s Dilemma game (PD), with the classical payoffs *π*: $T=5 > R=3 > P\,=$ $1 > S=0$^7,60^, synchronously with each peer *j* ∈ **F**_*i*_. The set of possible actions of agent *i* with agent *j* at time *t* is then given by ${{\bf{S}}}_{ij}^{t}$ = {*c*, *d*} (agents can cooperate, *c*, or defect, *d*), and the strategy **T**_*i*_ defines the behaviour of the agent in the game. As our primary focus is on different forms of indirect reciprocity, we thus consider six simple strategies (depicted in Fig. 1):

- *Unconditional Defection (UD)*: Always defects regardless of the behaviour of interaction partners, formally ∀*j*: ${{\bf{S}}}_{ij}^{t}=d$.
- *Unconditional Cooperation (UC)*: Always cooperates regardless of the behaviour of interaction partners, ∀*j*: ${{\bf{S}}}_{ij}^{t}=c$.
- *Tit for Tat (TFT)*: Reciprocates the last action of the interacting partner in the given dyadic relationship (it starts by defecting), formally ${{\bf{S}}}_{ij}^{t}={{\bf{S}}}_{ji}^{t-1}$. Similar results with a generally higher cooperation rate follow if we assume that TFT starts with cooperation (see Figs S1, S10 and S11).
- *Connected Reciprocity (CR)*: Reciprocates the last action of the interacting partner with a common connection, formally ${{\bf{S}}}_{ij}^{t}={{\bf{S}}}_{jz}^{t-1}$ for one randomly selected *z* ∈ **F**_*i*_ ∧ **F**_*j*_.
- *Unconnected Reciprocity (UR)*: Reciprocates the last action of the interacting partner with a connection of the latter, ${{\bf{S}}}_{ij}^{t}={{\bf{S}}}_{jz}^{t-1}$ for one randomly selected *z* ∈ **F**_*j*_.
- *Stern Judging (SJ)*: Rewards partners who cooperated with good partners with cooperation, partners who defected against good partners with defection, punishes partners who cooperated with bad partners with defection, and partners who defected with bad partners with cooperation. Formally:

1 $${{\bf{S}}}_{ij}^{t}=(\begin{array}{cc}{{\bf{S}}}_{jz}^{t-1} & {\rm{if}}\,\,{{\bf{S}}}_{zi}^{t-1}=c\\ \neg {{\bf{S}}}_{jz}^{t-1} & {\rm{if}}\,\,{{\bf{S}}}_{zi}^{t-1}=d\mathrm{.}\end{array}$$

If there is no previous action to observe, then the indirect reciprocity strategies act randomly with 50% chance of cooperation.

Agent-based simulations start with a predefined proportion of different type of strategies. Unless otherwise noted, an equal number of examined strategy types are present at the outset, each agent having the same probability of being any of the types. Simulations are run in discrete time periods. At each time step *t*, each agent *i* contemporaneously play the PD with each agent in his first order social neighborhood *j* ∈ **F**_*i*_. Agents of type UR and CR observing *defection* from the interacting partner give the partner another chance, *reacting to another one* of his previous actions, with probability *P*_*for*_ (i.e. *i* selects a new *z*′ playing ${{\bf{S}}}_{ij}^{t}={{\bf{S}}}_{jz^{\prime} }^{t-1}$).

At the end of the interaction phase, the *average payoff* ${\hat{\pi }}_{i}^{t}$ is calculated for each indvidual and compared with that of peers in the direct neighborhood (i.e. interaction partners). With probability *P*_*evo*_ the individual changed its strategy into the one of the best performing partner (copy-the-best update rule). In other terms:

2 $${{\bf{T}}}_{i}^{t+1}=(\begin{array}{cc}{{\bf{T}}}_{x}^{t} & {\rm{w}}{\rm{h}}{\rm{e}}{\rm{r}}{\rm{e}}\,\,x=\{j\,:j=argmax({\hat{\pi }}_{j}^{t})\,{\rm{\& }}\,j\in {{\bf{F}}}_{i}\}\,{\rm{w}}{\rm{i}}{\rm{t}}{\rm{h}}\,\,{P}_{evo}\\ {{\bf{T}}}_{i}^{t} & otherwise\end{array}$$

In case of ties, one of the individuals with the highest payoff is selected randomly. We run all simulations also with the alternative copy-the-better update rule, according to which the individual changed its strategy randomly into one that performed better in the immediate network neighborhood with probability *P*_*evo*_. Formally:

3 $${{\bf{T}}}_{i}^{t+1}=(\begin{array}{cc}{{\bf{T}}}_{x}^{t} & {\rm{w}}{\rm{h}}{\rm{e}}{\rm{r}}{\rm{e}}\,\,x=\{j\,\,:{\hat{\pi }}_{j}^{t} > {\hat{\pi }}_{i}^{t}\,{\rm{\& }}\,j\in {{\bf{F}}}_{i}\}\,{\rm{w}}{\rm{i}}{\rm{t}}{\rm{h}}\,{P}_{evo}\\ {{\bf{T}}}_{i}^{t} & otherwise.\end{array}$$

Again, in case of ties one agent is selected randomly. The parameter *P*_*evo*_ describes the speed of evolution that is generally considered to favor defection^61^. The intra-step dynamics, repeated synchronously for each agent *i*, is described by Algorithm 1.

∀*i* & ∀*j* ∈ **F**_*i*_: Let *i* and *j* select their choices in the PD according to ${{\bf{T}}}_{i}^{t}$ and to **S**^*t*−1^

∀*i*: compute $${\hat{\pi }}_{i}^{t}$$

∀*i*:

   Observe the average payoffs of period *t* for each agent *j* ∈ **F**_*i*_

   Adopt the strategy in $j\in {{\bf{F}}}_{i}^{t-1}$ that yields the maximum/higher average payoff (with probability *P*_*evo*_)

**Algorithm 1**. Intra-step dynamics, repeated at each time step *t*.

Simulations lasted until all agents have started to follow the same strategy or 100,000 periods have passed. Note that some simulations did not reach convergence. Moreover, the dominance of one strategy type does not mean that there are no further changes in behavior. For instance, a homogeneous set of UR players can still act differently towards different partners. Asymptotic behaviour for populations of each strategy are reported in Section 12 of SI. All code and data reported in this article and in the supplementary material are available online^62^.

## Results

### Direct and indirect reciprocity

The significance of different forms of reciprocity might vary and one form could potentially drive out the feasibility of others. As shown in the computer tournament results of Axelrod^1,7^ and in the comparative study of Roberts^4^ in the Prisoner’s Dilemma, TFT comes to dominate the population in almost every simulation when compared with indirect reciprocity strategies. The strength of direct reciprocity is testified by the results in Fig. S1 where we manipulate the initial proportion of TFT strategies, showing that this strategy can become prevalent even when initially played by a small share of individuals. For this reason, the strength of direct reciprocity overshadows the differential performance of indirect reciprocity strategies.

### Relative performance of indirect reciprocity strategies

**Figure 2:**
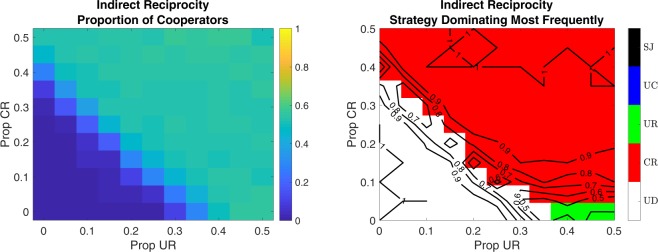
Effect of the Initial Proportion of UR and CR strategies. *Left Panel*: Final average proportion of cooperators. *Right Panel*: Strategy that is dominating more often (colors) and proportion of simulations dominated by that strategy (lines). For each parameter combination 100 simulations were run on E-R random networks of 240 individuals with *λ* = 0.10. *P*_*evo*_ = 0.05 and *P*_*for*_ = 0. The initial proportion of UR is indicated on the x-axis and the initial proportion of CR on the y-axis. The remaining population is initialized as equally divided among UC, UD, and SJ strategies (TFT is absent).

We compare the performance of indirect reciprocity strategies under various initial proportions of Connected Reciprocity (CR) and Unconnected Reciprocity (UR), while keeping the rest of the initial population equally divided among the remaining strategies. We explore a large set of possible initial proportions for these strategies. Results are summarized in Figs 2, S2, and S3. In the shadow of the success of direct reciprocity, the CR strategy is more successful than UR in the majority of cases, even when UR is more represented initially (Fig. 1). The heatmaps show that above a certain threshold of their presence, a comparable high level of cooperation is achieved irrespective of the precise initial proportions of UR and CR strategies, considering the copy-the-best strategy update rule. This result is not generalizable to the copy-the-better strategy update rule, where a larger presence of CR strategies at the outset results in significantly higher cooperation rates (Figs S4–S5). The level of cooperation reaches its peak when half of the players follow the CR strategy, a quarter of the population plays the UR strategy, and other strategies are also represented. An exception to the general pattern is the case when CR strategies are absent and the UR strategy is followed by half of the initial population. In this exceptional case, full cooperation is reached.

Simulations always ended up with the extinction of the Stern Judging (SJ) strategy. This is remarkable because similarly to CR, SJ is based on social closure, but it relies on even more precise information. It does not purely reward cooperation in closed circles, but also evaluates if cooperation was appropriate as it rewarded a cooperative partner or not. In this way, SJ is not purely a second order strategy that implies enforcement of norms of cooperation, but it is a third order strategy that benefits those partners who were policing the enforcement of cooperative norms properly. Due to its complexity, however, SJ could easily be trapped in cycles of misinterpretations. SJ does not achieve any success because it strongly relies on the establishment of mutual cooperative relations between alters and on the lack of mistakes and randomness at the outset. The weak role of SJ in this context is confirmed by the fact that no major qualitative difference are observed in its absence (Fig. S3). This departure from the results of other papers^35^ hints to a weakness of complex strategies, such as SJ, when interactions are localized.

**Figure 3:**
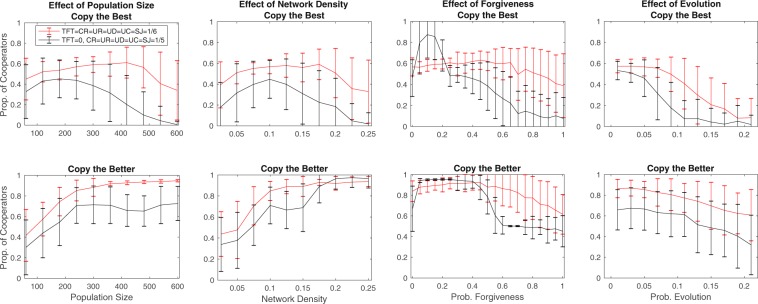
Effect of population size *N* (First Panel), network density *λ* (Second Panel), forgiveness *P*_*for*_ (Third Panel), and *P*_*evo*_ (Fourth Panel) on the final proportion of cooperation. Results for copy-the-best evolutionary update (Upper Panels) and for copy-the-better evolutionary update (Lower Panels). *N* = 240 for each parameter value.

We also compare the performance of indirect reciprocity strategies when direct reciprocity is excluded from the population. The levels of cooperation attained in absence of TFT are generally lower than those obtained when direct reciprocity becomes dominant (Fig. 3). Connected Reciprocity overrules Unconnected Reciprocity showing the power of accountability in closed social circles (Figs S6–S9).

We test the robustness of these results in an alternative structural configuration: a regular lattice with a homogenous degree and the same number of closed triads for each agent. We create cliques of four nodes, and exactly one tie to a non-clique member in a regular way (Fig. S12). Results in Figs S13–S14 show an advantage of the CR strategy that is somewhat less overwhelming than in E-R networks. In the regular lattice network, when the UR strategy is present in a large number at the outset, it is able to gain dominance in the population. The reason for this is that in a regular lattice the UR strategy also channels largely embeddeded and hence reliable information.

### The impact of population size

Contrary to intuitive arguments, increasing the population size does not turn down the success of direct reciprocity in favor of indirect reciprocity (Fig. 3). TFT, when present, produces higher levels of cooperation and becomes dominant more often than indirect reciprocity strategies. In the case of the copy-the-best strategy update, a saturation point for cooperation emerges when increasing population size. Moreover, large populations under this evolutionary rule do not produce cooperation at all if TFT is absent, as UD becomes dominant.

The effect of population size on cooperation is largely different when we consider the copy-the-better strategy update. This evolutionary update rule provides more favorable conditions for cooperation than the copy-the-best rule in general^63^, because it keeps conditional cooperation alive, while it does not help the proliferation of overly successful defection strategies who benefit from cheating with multiple partners. Simulation runs in which TFT is present reach full cooperation with population size over 300. It is interesting to note that cooperation rates are lower for smaller than for larger population sizes. Hence, the slowlier adoption rule works more efficiently in a larger network where it has more time to spread (Figs 3 and S8).

### Direct reciprocity with limited memory

The dominance of direct reciprocity depends largely on the assumption that TFT recalls perfectly the previous actions of its partners, so that discriminatory practices can be applied to all partners. To test the robustness of direct reciprocity success against indirect reciprocity, we relax the assumption of its perfect memory. We assume that agents playing TFT remember the last action of the partner with a given probability. If they have perfect recall (maximum efficiency), then they reciprocate the previous action of all partners. Otherwise, they may forget the previous action of some partners and revert to their basic behaviour.

**Figure 4:**
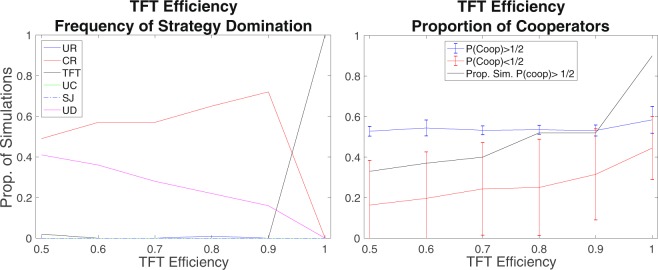
Effect of TFT efficiency (perfectness of recall) on which strategies gain absolute dominance and on the proportion of cooperation. Simulation runs with imperfect recall could clearly be separated into two scenarios. The Right Panel reports the proportion of simulations that end up in each scenario (i.e. with a final proportion of cooperators above and below 1/2), and the proportion of cooperation in each scenario. Results are provided for an initialization where the population is equally divided among the types of agents. Results come from 100 simulations for each parameter combination for E-R random networks of 240 individuals with *λ* = 0.10. *P*_*evo*_ = 0.05; *P*_*for*_ = 0. The rule of strategy update is the copy-the-best strategy.

Results show that TFT is clearly vulnerable to a memory constraint (Figs 4 and S10–S11). The domination of direct on indirect reciprocity strongly depends on perfect recall by the TFT rule. As soon as the perfectness is relaxed, the TFT strategy loses dominance in the population and it is substituted in his role by Connected Reciprocity (CR). Concerning cooperation, two equilibria emerge: one where cooperation ends up being played half of the times, and one where the whole population defects. The memory constraints of TFT generate a situation that ranks the CR strategy clearly higher than UR. Given the imperfectness of TFT, the direct consequences of Connected Reciprocity for all members of the triad makes this strategy viable. In contrast, Unconnected Reciprocity proves to be inefficient as its good intentions without the enforcement of local social control are easily exploited with defection. These results are independent from the evolutionary update rule applied and from TFT’s default action (Figs S10–S11).

### Network density

Network density has a non-monotonous effect on the success of Connected Reciprocity and also on the proportion of cooperation in the case of the copy-the-best update rule. High densities allow the acquisition of more complete information. In these conditions, the best strategies are those that exploit the most partners with defection^64^. This is different when the copy-the-better update rule is used, where a larger density implies more cooperation (Fig. 3). The number of closed triangles is larger in a denser network that creates place for a better application and control of the Connected Reciprocity strategy (further details in SI Fig. S9).

### The importance of forgiveness

When defection is observed with a third party, indirect reciprocity strategies could forgive this action and check the opponent’s behavior in another encounter. Cooperation increases when forgiveness of this kind is introduced, particularly when TFT is missing from the initial population (Fig. 3). Just like for TFT in the dyad^9,10,65,66^, some forgiveness helps to break the vicious retaliation circles of defection in a triad of CR strategies. High levels of forgiveness, though, are problematic for cooperation, as they imply the neglect of relevant information and constrain effective punishment of a defecting partner. The level of cooperation is higher in the copy-the-better rule for all levels of forgiveness and it is plateaud at a very high level of cooperation for a larger range of forgiveness values.

### Summary of results

In summary, we analyze the relative effectiveness of variants of indirect reciprocity in the presence and in the absence of TFT. Motivated by the sociological debate on the nature of social capital^38,41–43,50,67^, we focus on the relative efficiency of Connected and Unconnected Reciprocity strategies. Connected Reciprocity benefits from social closure and relies on information from those individuals who are also tied to the partner. Unconnected Reciprocity gains information from anyone and reciprocates the action of the partner towards the source of information. In this way, our main question origins in the dilemma whether indirect reciprocity is able to operate efficiently due to cohesive aspects of social capital in closed circles or because it utilizes any available information about the partner, also from those who are not direct interaction partners.

When direct reciprocity is present, it tends to reach evolutionary success in the Prisoner’s Dilemma on simple networks. This is also the case in larger populations where - according to some arguments - direct reciprocity is supposed to be replaced by indirect reciprocity. The impact of population size itself depends on the evolutionary update rule considered. On one hand, if individuals copy only their best performing neighbors, then large populations sustain a lower proportion of cooperation than small populations. On the other hand, if individuals are satisfied with updating to a neighbor strategy that simply performs better than their own, then cooperation is favored even in larger populations. The well-known cooperation boosting effect of the copy-the-better update rule is crystallized in larger populations that do not let cooperative strategies disappear suddenly. Our results show that the Tit For Tat (TFT) strategy prevails, unless memory constraints are introduced. In the presence of the latter, indirect reciprocity strategies dominate and establish cooperation.

Results show that indirect reciprocity strategies are able to maintain cooperation, but Connected Reciprocity is a better performer. This is a robust result which characterizes the situation when direct reciprocity suffers from imperfect recall, and also the case when direct reciprocity is excluded from the initial set of strategies.

## Discussion

The evolution of cooperation is one of the fundamental problems of human social organization^7,68^. Simple reciprocal and trigger strategies have shown to be prevalent in this process^3,69,70^. Our results further underline the power of direct reciprocity (Tit For Tat) in evolutionary contests of the Prisoner’s Dilemma and for the evolution of cooperation. In line with the findings of Roberts^4^, direct reciprocity outperforms indirect reciprocity because it can immediately identify and punish defections of previous partners.

The perfect tailoring of reciprocation, however, is constrained by individual memory capacities. Our results show that direct reciprocity in fact loses dominance as imperfect recall is introduced for TFT. It is important to emphasize that not population size per se, but individual memory constraints are responsible for the decline of direct reciprocity. This potentially implies that cognitive constraints that had to be complemented with communication could have helped hominid groups to achieve impersonal cooperation.

When direct reciprocity fails to gain dominance in our model, the relative strength of indirect reciprocity strategies is considered. The basic idea behind indirect reciprocity strategies is that individuals are able to observe interactions in which they are not directly involved^36^. In human societies with the facilities of advanced human communication, and gossip in particular, direct observation is not necessary and the feasibility of indirect reciprocity strategies is further improved^71–74^.

There is a quite large extent of ambiguity in the literature about what indirect reciprocity means exactly. While indirect reciprocity has been defined in various ways, we focus on a fundamental difference in what source of information the strategy accounts for. According to Unconnected Reciprocity, any third-party information could be useful for conditioning behavior against an interaction partner. Due to information flow in open triads, Unconnected Reciprocity might produce global dissemination of behavior more easily. This is the key aspect of social capital characterized by the presence of structural holes^50^, and weak, far-reaching ties^48^. In contrast, Connected Reciprocity only conditions cooperation on the information from mutual partners and has immediate positive externalities. This strategy benefits from social capital that is conceptualized differently: it builds on the reliability and accountability of closed and cohesive microstructures.

We show that Unconnected Reciprocity that benefits from information from indirectly related individuals loses the competition with Connected Reciprocity that builds on the strength of social circles, substantiating the relevance of social capital in closed triads. In game theoretic terms, local play and information in cohesive micro networks create a correlation device that allows for the clustering of cooperators that establish the success of Connected Reciprocity^75^. Closed triads secure the chances of indirect reciprocity by allowing retaliation that has factual consequences for each member of the triad in one or two steps. In contrast, Unconnected Reciprocity relies on information from individuals who were not and will not be interaction partners. As play never happens between the source and the recipient of information, defection is a better response even to the cooperative act of the partner to the third party. Observation of behavior of the partner with an unrelated third party therefore is insufficient to enforce a circle of cooperation, as the third party could always exploit the benefits of the structural hole^50^. Despite the superiority of Connected Reciprocity, cooperation has been achieved also with its Unconnected counterpart, due to the fact that at least the partner in the brokerage position is constrained by indirect punishment from its other contacts playing the UR strategy. The relative success of Connected Reciprocity benefiting from closed triads can be linked to the empirical observation that social networks tend to be small world structures^76^ that provide even more favorable conditions for Connected Reciprocity.

Note that the viability of indirect reciprocity strategies in social dilemmas relies on strong assumptions that communication is frequent and factual^14,26,33,37,77–79^. For indirect reciprocity to work, the reliability of information has to be known with a certain accuracy^12,33,78^. When honesty is not hard-wired, indirect reciprocity cannot prevail: the possibility of deception might nullify all model results^33^.

## Electronic supplementary material

Supplementary Information
