# Publisher Correction: Social Closure and the Evolution of Cooperation via Indirect Reciprocity

**DOI:** 10.1038/s41598-019-43810-6

**Published:** 2019-10-30

**Authors:** Simone Righi, Károly Takács

**Affiliations:** 1 Department of Computer Science, University College London, London, WC1E 6EA United Kingdom; 2 Hungarian Academy of Sciences, MTA TK “Lendület” Research Center for Educational and Network Studies (RECENS), Budapest, 1097 Hungary

Correction to: *Scientific Reports* 10.1038/s41598-018-29290-0, published online 24 July 2018

In the original version of this Article, Károly Takács was incorrectly affiliated with ‘Department of Computer Science, University College London, London, WC1E 6EA, United Kingdom’. The correct affiliation is listed below.

Hungarian Academy of Sciences, MTA TK “Lendület” Research Center for Educational and Network Studies (RECENS), Budapest, 1097, Hungary

This error has now been corrected in the PDF and HTML versions of the Article.

