# Supplementary material for: Social Closure and the Evolution of Cooperation via Indirect Reciprocity
Source: Sci Rep. 2018 Jul 24;8:11149. doi: 10.1038/s41598-018-29290-0 (PMC6057955; doi:10.1038/s41598-018-29290-0)
Supplement: Supplementary file 1 — Supplementary Information [file 41598_2018_29290_MOESM1_ESM.pdf]

Social Closure and the Evolution of Cooperation via Indirect Reciprocity

Simone Righi*† Károly Takács ‡

*MTA TK "Lendület" Research Center for Educational and Network Studies (RECENS), Hungarian Academy of Sciences. Mailing address: Tóth Kálmán utca 4-6, 1097 Budapest, Hungary. Email: simone.righi@tk.mta.hu.

†Department of Computer Science, University College London Email: s.righi@ucl.ac.uk

‡MTA TK "Lendület" Research Center for Educational and Network Studies (RECENS), Hungarian Academy of Sciences. Mailing address: Tóth Kálmán utca 4-6, 1097 Budapest, Hungary. Email: takacs.karoly@tk.mta.hu

1 Effect of the Initial Proportion of TFT

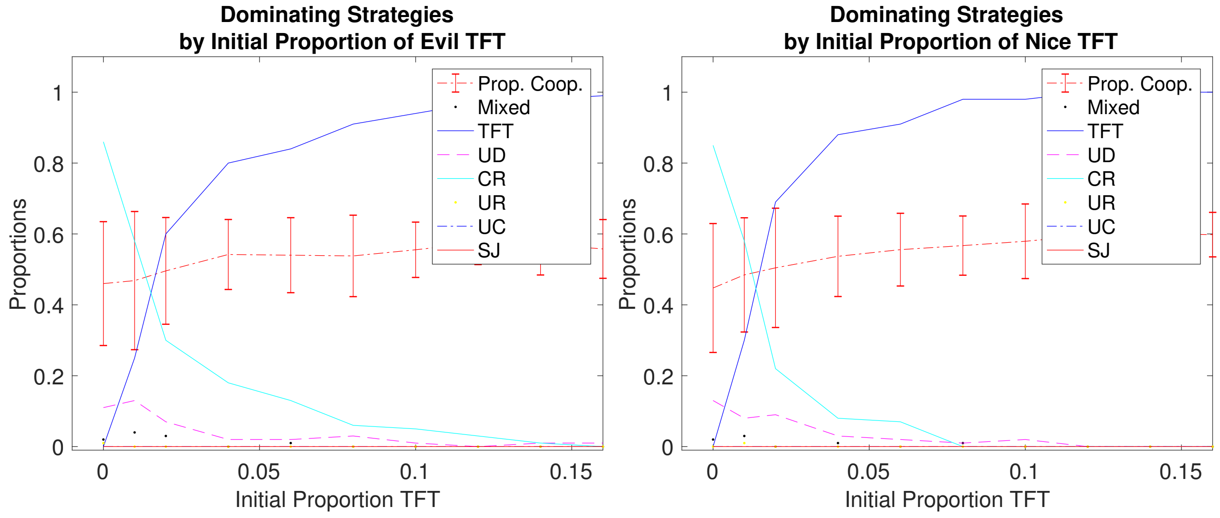

Figure S1: The effect of the initial proportion of agents playing the TFT strategy on the final proportion of cooperation, and on the dominating strategy types. *Notes:* Results are averages and standard deviations of 100 simulations for each parameter combination for E-R random networks of 240 individuals ($\lambda = 0.10$); with $P_{evo} = 0.05$; $P_{err} = 0$; $P_{for} = 0$. The initial proportion of TFT agents is indicated on the x-axis, while the remaining population is is initialized as equally divided among the other 5 types of agents. The evolutionary update rule is copy-the-best. A strategy is defined as dominating when is adopted by at least 90% of agents at the end of the simulation. **Left Panel**: Results for evil TFT (the strategy starts off by defecting). **Right Panel**: Results for nice TFT (the strategy starts off by cooperating).

2 Performance of Indirect Reciprocity Strategies

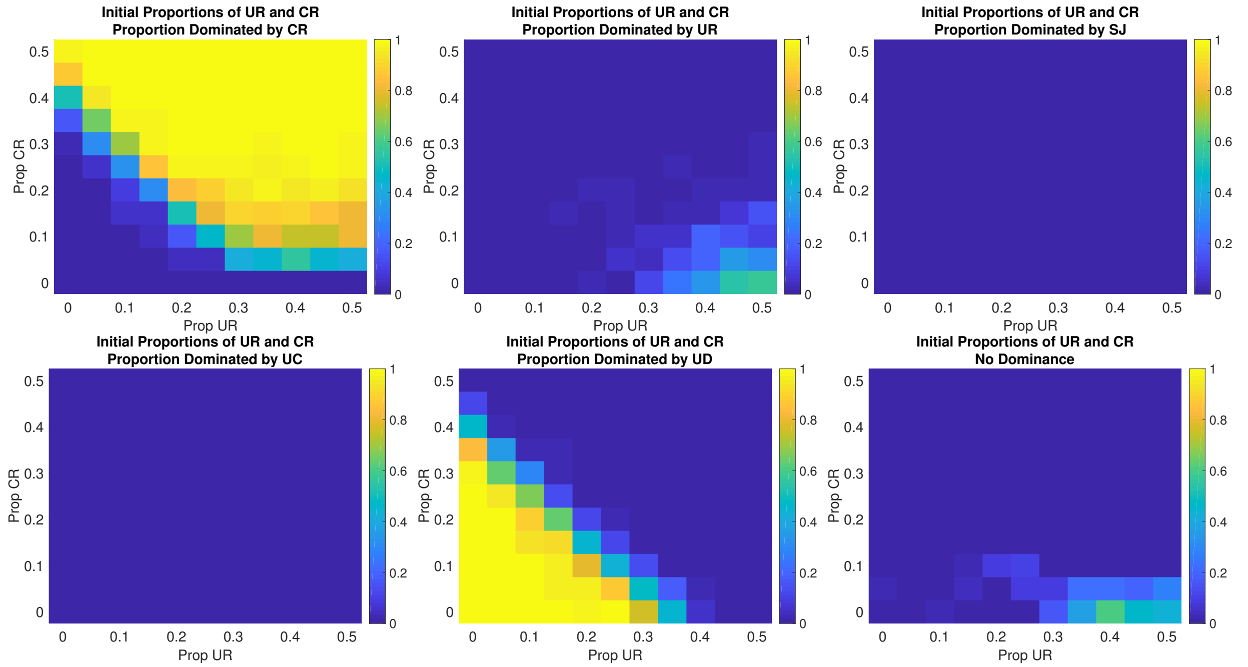

Figure S2: Effect of the initial proportion of UR and CR strategies on the final proportion of simulations dominated by each strategy. *Notes:* The evolutionary update rule is copy-the-best. Domination is defined as one strategy being adopted by at least 90% of agents at the end of the simulation. Results report the proportion of simulations dominated by each strategy. For each parameter combination 100 simulations are run on a E-R random networks of 240 individuals ($\lambda = 0.10$); with $P_{evo} = 0.05$; $P_{err} = 0$; $P_{for} = 0$. The initial proportion of UR strategies is indicated on the x-axis, and the initial proportion of CR strategies are on the y-axis, the remaining population is initialized as equally divided among UC, UD and SJ (TFT is absent). *Top Left Panel*: Proportion of simulations dominated by CR, *Top Central Panel*: Proportion of simulations dominated by UR, *Top Right Panel*: Proportion of simulations dominated by SJ, *Bottom Left Panel*: Proportion of simulations dominated by UC, *Bottom Central Panel*: Proportion of simulations dominated by UD, *Bottom Right Panel*: Proportion of simulations with no dominating strategy.

3 Performance of Indirect Reciprocity Strategies without SJ

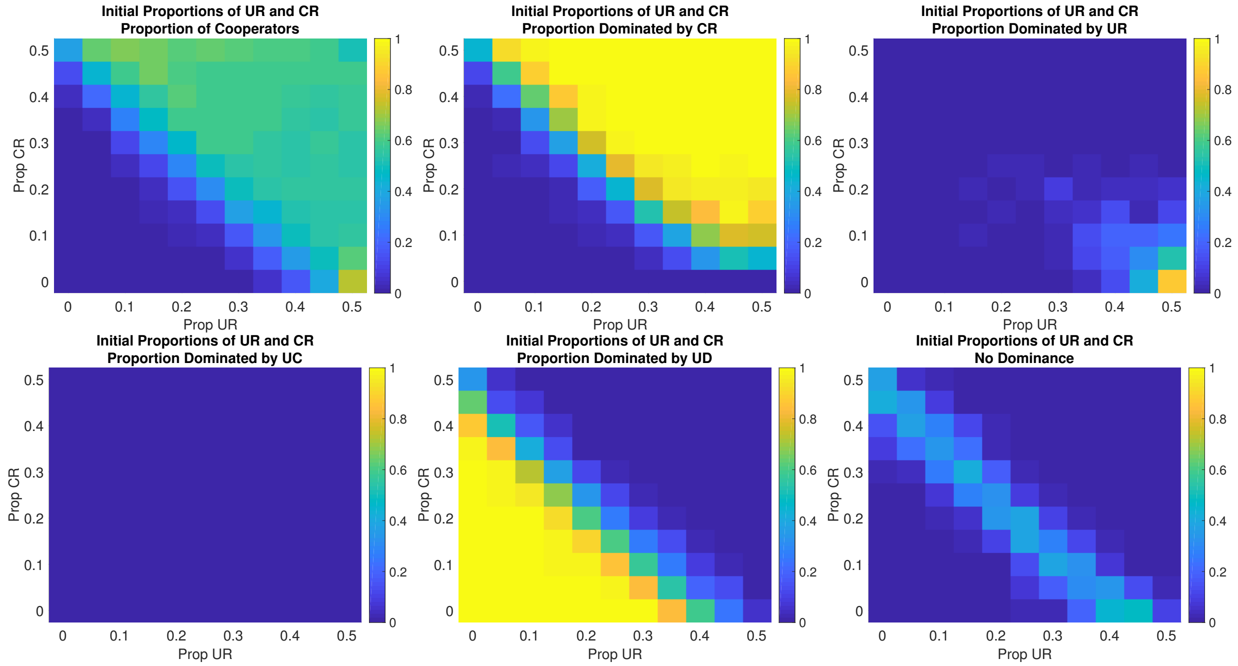

Figure S3: Effect of the Initial Proportion of UR and CR on the final proportion of simulations dominated by each strategy and on prevalence of cooperative actions. *Notes:* The evolutionary update rule is copy-the-best. Domination is defined as one strategy being adopted by at least 90% of agents at the end of the simulation. Results report the proportion of simulations dominated by each strategy. For each parameter combination 100 simulations are run on a E-R random networks of 240 individuals ($\lambda = 0.10$); with $P_{evo} = 0.05$ and with $P_{for} = 0$. The initial proportion of UR is indicated on the x-axis the initial proportion of CR on the y-axis, the remaining population is initialized as equally divided among UC and UD (TFT and SJ are absent). *Top Left Panel*: Proportion of cooperators, *Top Central Panel*: Proportion of simulations dominated by CR, *Top Right Panel*: Proportion of simulations dominated by UR, *Bottom Left Panel*: Proportion of simulations dominated by UC, *Bottom Central Panel*: Proportion of simulations dominated by UD, *Bottom Right Panel*: Proportion of simulations with no dominating strategy

4 Performance of Indirect Reciprocity Strategies - Copy-the-Better Update Rule

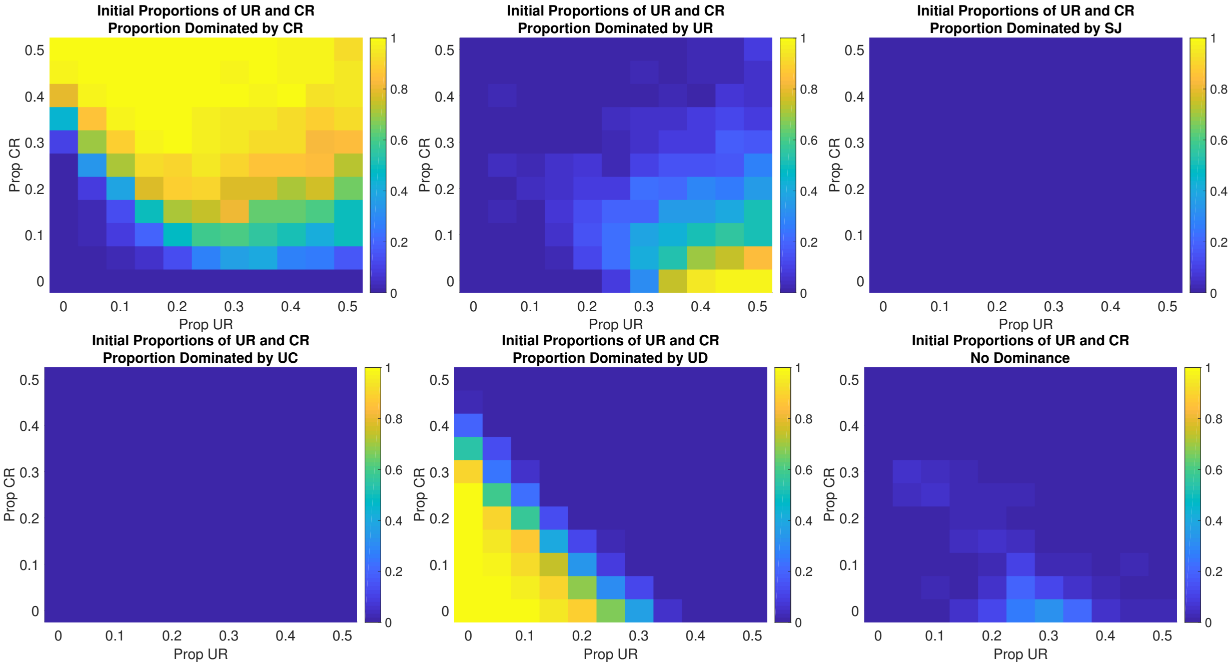

Figure S4: Effect of the initial proportion of UR and CR strategies on the final proportion of simulations dominated by each strategy. *Notes:* The evolutionary update rule is copy-the-better. Domination is defined as one strategy being adopted by at least 90% of agents at the end of the simulation. Results report the proportion of simulations dominated by each strategy. For each parameter combination 100 simulations are run on a E-R random networks of 240 individuals ($\lambda = 0.10$); with $P_{evo} = 0.05$; $P_{err} = 0$; $P_{for} = 0$. The initial proportion of UR strategies is indicated on the x-axis, and the initial proportion of CR strategies are on the y-axis, the remaining population is initialized as equally divided among UC, UD and SJ (TFT is absent). *Top Left Panel*: Proportion of simulations dominated by CR, *Top Central Panel*: Proportion of simulations dominated by UR, *Top Right Panel*: Proportion of simulations dominated by SJ, *Bottom Left Panel*: Proportion of simulations dominated by UC, *Bottom Central Panel*: Proportion of simulations dominated by UD, *Bottom Right Panel*: Proportion of simulations with no dominating strategy.

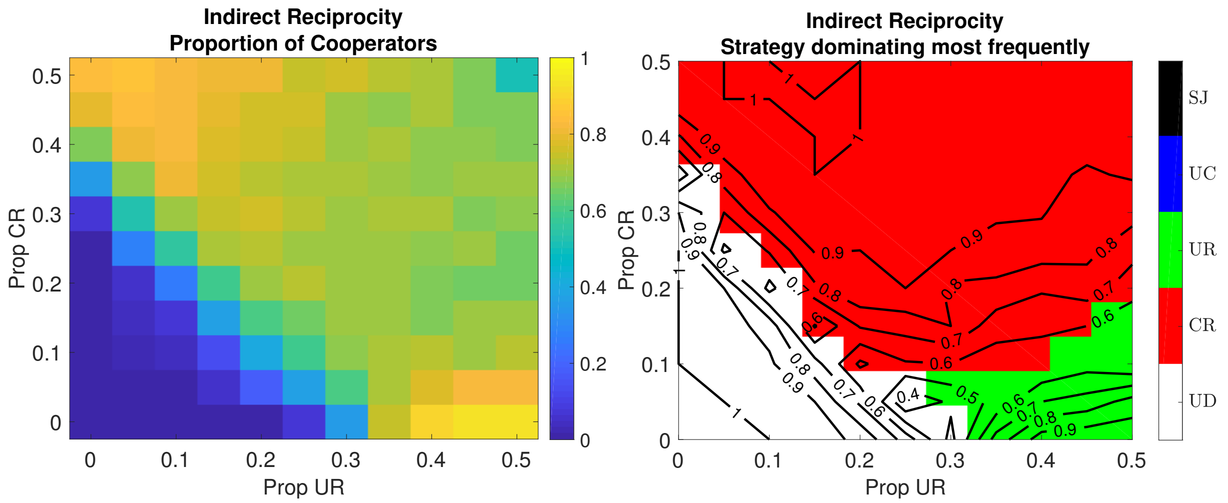

Figure S5: Effect of the Initial Proportion of UR and CR. The evolutionary update rule is copy-the-better. *Left Panel*: Final average proportion of cooperators. *Right Panel*: Type of agent that dominates more often (colors) and proportion of simulations dominated by that strategy (lines). For each parameter combination 100 simulations were run on E-R random networks of 240 individuals with $\lambda = 0.10$. $P_{evo} = 0.05$ and $P_{for} = 0$. The initial proportion of UR is indicated on the x-axis and the initial proportion of CR on the y-axis. The remaining population is initialized as equally divided among UC, UD, and SJ strategies (TFT is absent).

5 The Importance of Forgiveness

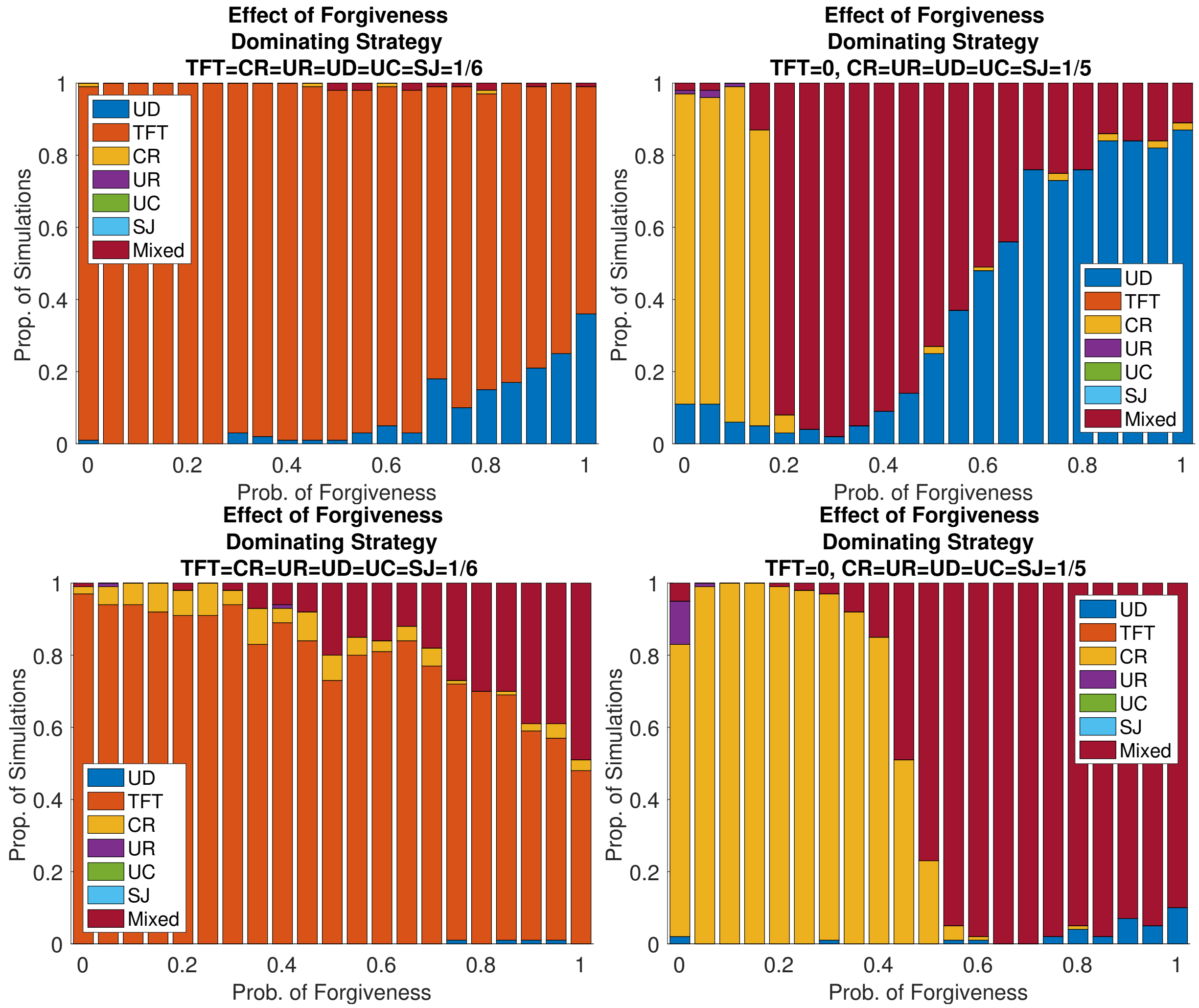

Figure S6: Effect of P_{for} on the proportion of simulations where more than 90% of agents are of each given type at the end of the simulation periods. *Notes:* Results proportions of 100 simulations for each parameter combination for E-R random networks of 240 individuals ($\lambda = 0.10$) and with $P_{evo} = 0.05$. Results are provided for two different population initialization. *Left Panels*: the population is equally divided among the six types of strategies. The TFT is the Evil version. *Right Panels*: TFTs are absent and the population equally divided among the remaining five type of strategies. *Top Panels*: The evolutionary update rule is copy-the-best. *Bottom Panels*: The evolutionary update rule is copy-the-better.

6 Speed of Evolution

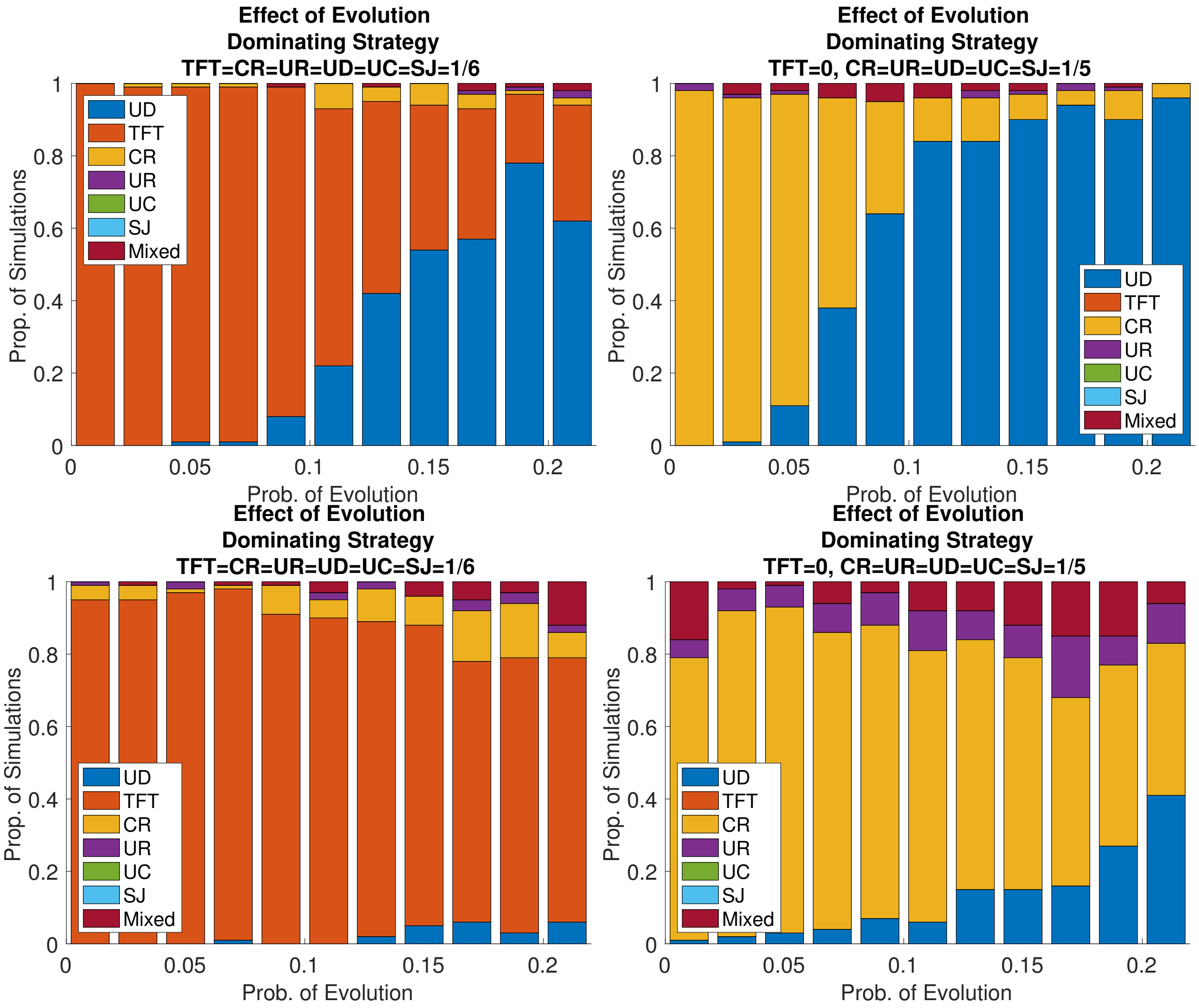

Figure S7: Effect of P_{evo} on the proportion of simulations where more than 90% of agents are of each given type at the end of the simulation periods. *Notes:* Results proportions of 100 simulations for each parameter combination for E-R random networks of 240 individuals ($\lambda = 0.10$) and with $P_{for} = 0$. Results are provided for two different population initialization. *Left Panels*: the population is equally divided among the six types of strategies. The TFT is the Evil version. *Right Panels*: TFTs are absent and the population equally divided among the remaining five type of strategies. *Top Panels*: The evolutionary update rule is copy-the-best. *Bottom Panels*: The evolutionary update rule is copy-the-better.

7 The Impact of Population Size

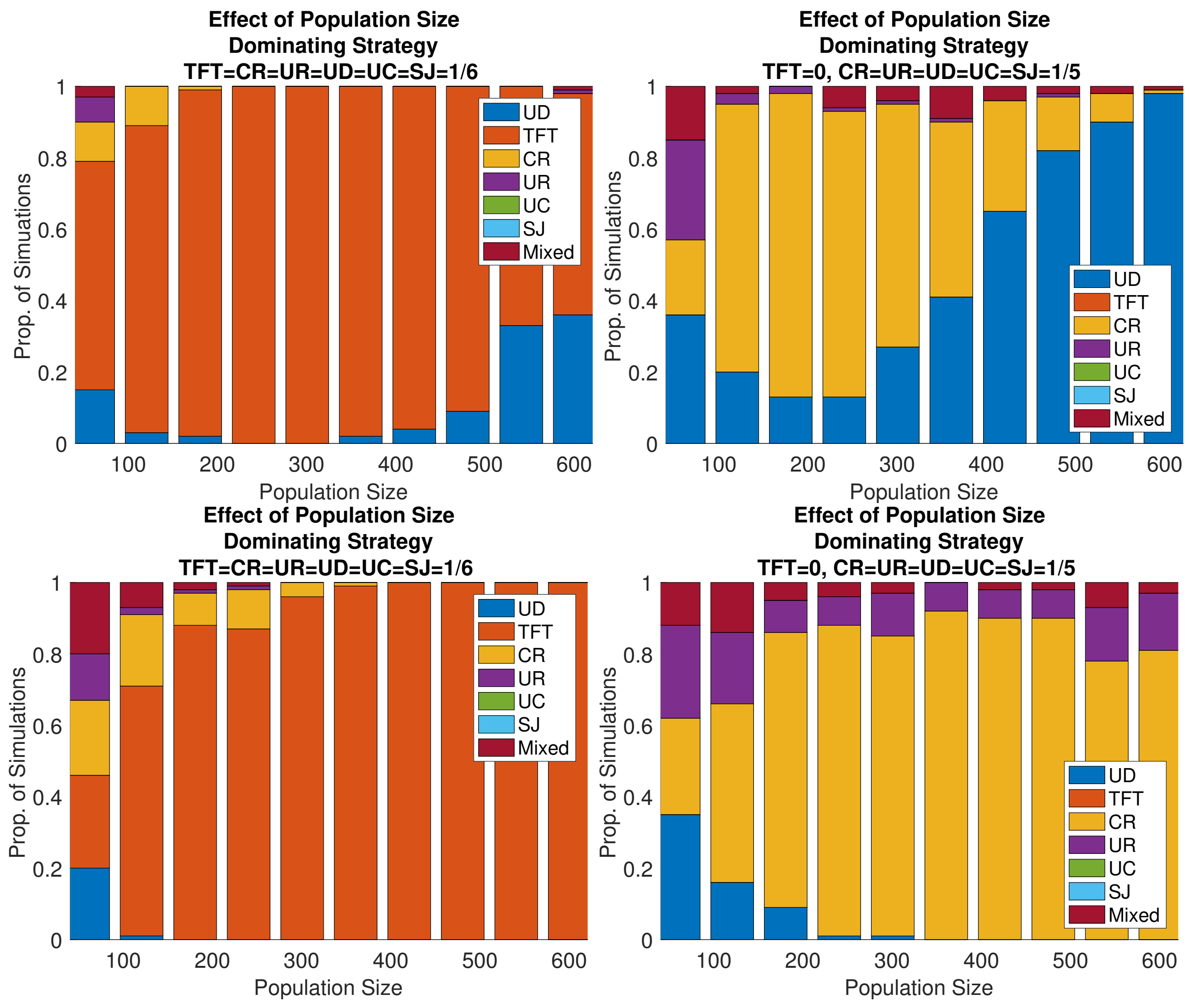

Figure S8: Effect of Population Size on the proportion of simulations where more than 90% of agents are of each given type at the end of the simulation periods. *Notes:* Results proportions of 100 simulations for each parameter combination for E-R random networks of 240 individuals ($\lambda = 0.10$), with $P_{for} = 0$ and $P_{evo} = 0.05$. Results are provided for two different population initialization. *Left Panels*: the population is equally divided among the six types of strategies. The TFT is the Evil version. *Right Panels*: TFTs are absent and the population equally divided among the remaining five type of strategies. *Top Panels*: The evolutionary update rule is copy-the-best. *Bottom Panels*: The evolutionary update rule is copy-the-better.

8 The Impact of Network Density

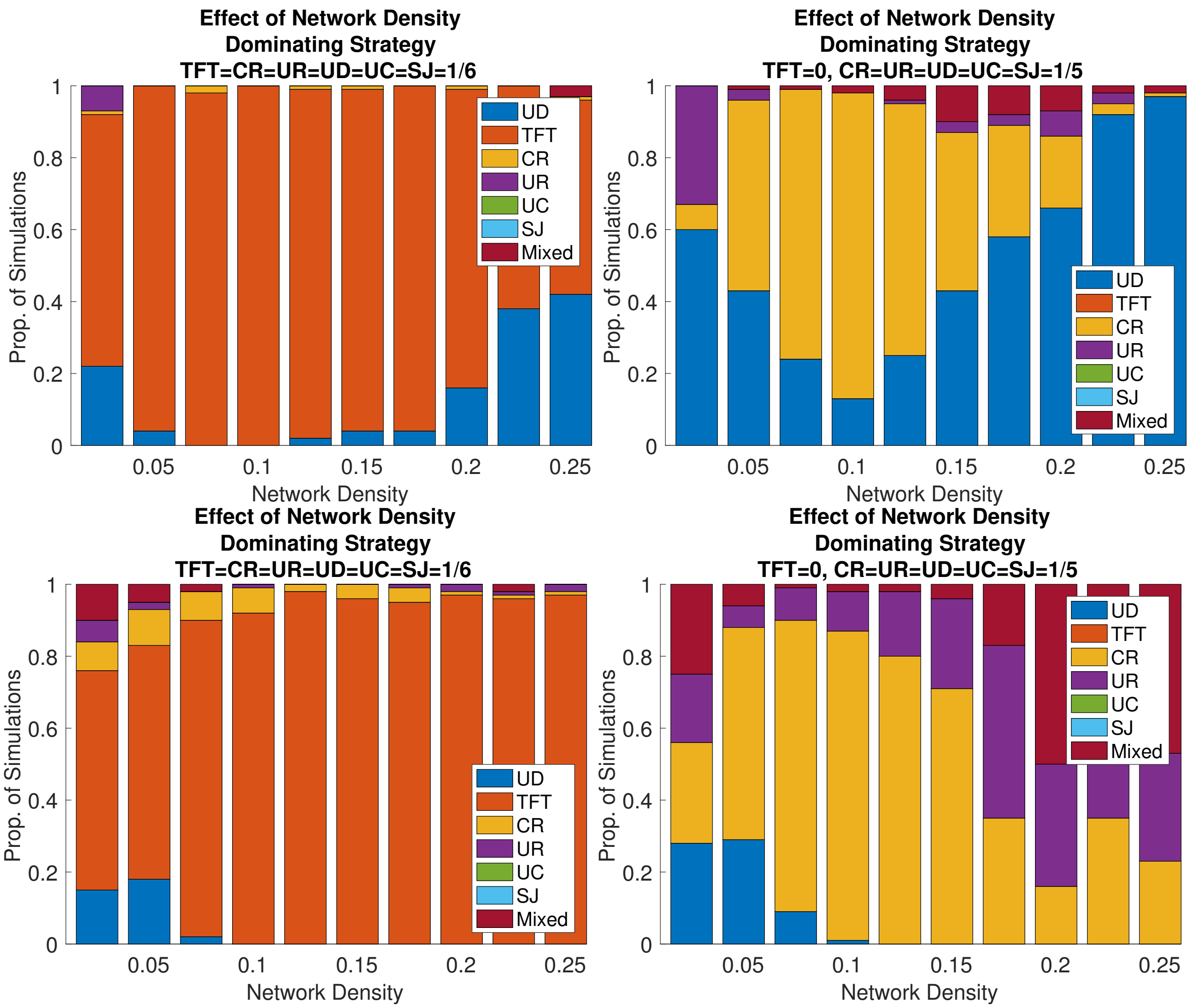

Figure S9: Effect of Network Density (d) on the proportion of simulations where more than 90% of agents are of each given type at the end of the simulation periods. *Notes:* Results proportions of 100 simulations for each parameter combination for E-R random networks of 240 individuals, with $P_{for} = 0$ and $P_{evo} = 0.05$. Results are provided for two different population initialization. *Left Panels*: the population is equally divided among the six types of strategies. The TFT is the Evil version. *Right Panels*: TFTs are absent and the population equally divided among the remaining five type of strategies. *Top Panels*: The evolutionary update rule is copy-the-best. *Bottom Panels*: The evolutionary update rule is copy-the-better.

9 The Impact of TFT Efficiency (Perfectness of Memory)

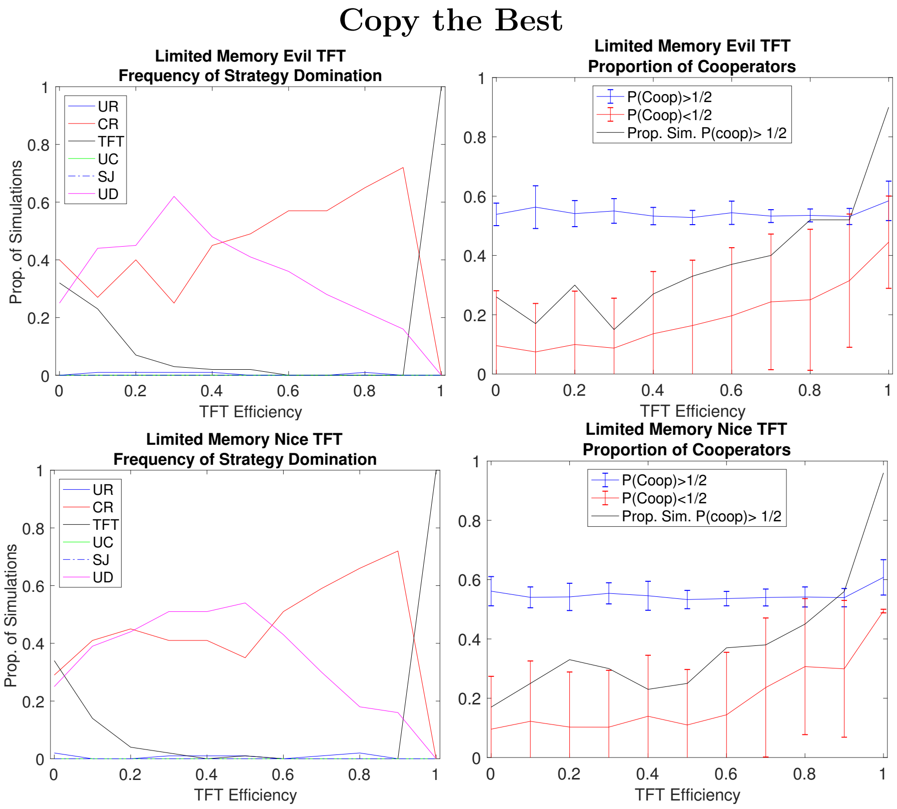

Figure S10: Effect of TFT imperfect memory on the proportion of simulations where more than 90% of agents are of each given type at the end of the simulation periods (*Left Panels*) and on the prevalence of cooperative acts (*Right Panels*). *Notes:* Results are provided for an initialization where the population is equally divided among the 6 types of agents. Results come from 100 simulations for each parameter combination, and the levels of cooperation report averages and standard deviations. The network is a E-R random networks of 240 individuals ($\lambda = 0.10$) ; $P_{evo} = 0.05$; $P_{for} = 0$. Different probabilities of remembering correctly the past action are considered between 0% and 100% in steps of 10%. *Top Panels*: the TFT strategy is "Evil" (i.e. starts by defecting). *Bottom Panels*: the TFT strategy is "Nice" (i.e. starts by cooperating). In all cases the evolutionary rule is **copy the best**.

Copy the Better

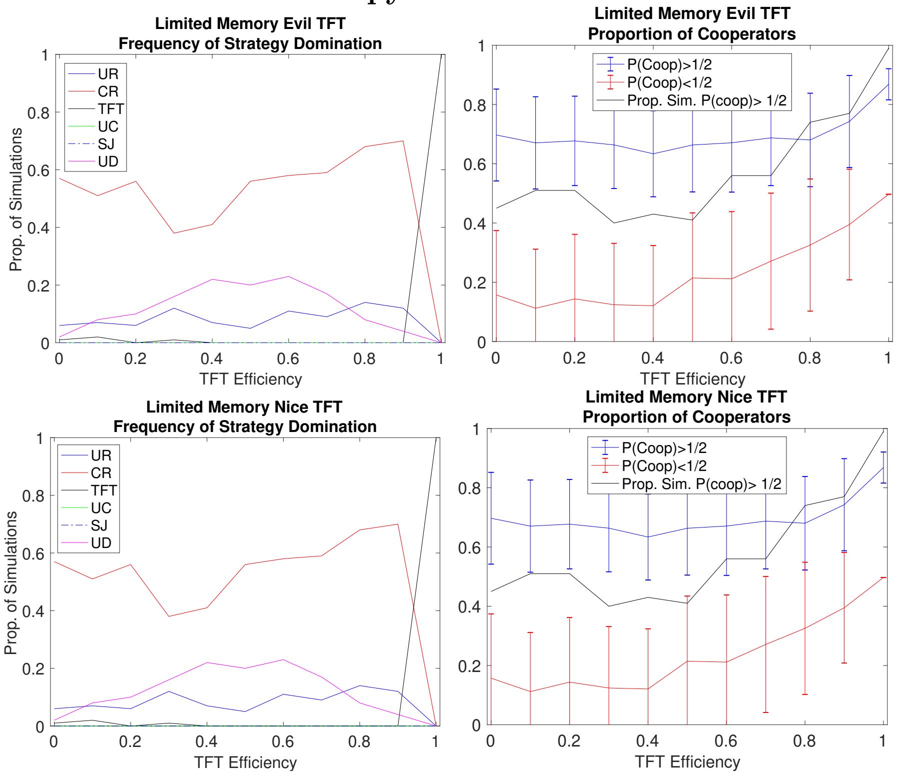

Figure S11: Effect of TFT imperfect memory on the proportion of simulations where more than 90% of agents are of each given type at the end of the simulation periods (*Left Panels*) and on the prevalence of cooperative acts (*Right Panels*). *Notes:* Results are provided for an initialization where the population is equally divided among the 6 types of agents. Results come from 100 simulations for each parameter combination, and the levels of cooperation report averages and standard deviations. The network is a E-R random networks of 240 individuals ($\lambda = 0.10$) ; $P_{evo} = 0.05$; $P_{for} = 0$. Different probabilities of remembering correctly the past action are considered between 0% and 100% in steps of 10%. *Top Panels*: the TFT strategy is "Evil" (i.e. starts by defecting). *Bottom Panels*: the TFT strategy is "Nice" (i.e. starts by cooperating). In all cases the evolutionary rule is **copy the better**.

10 Lattice Network Structure

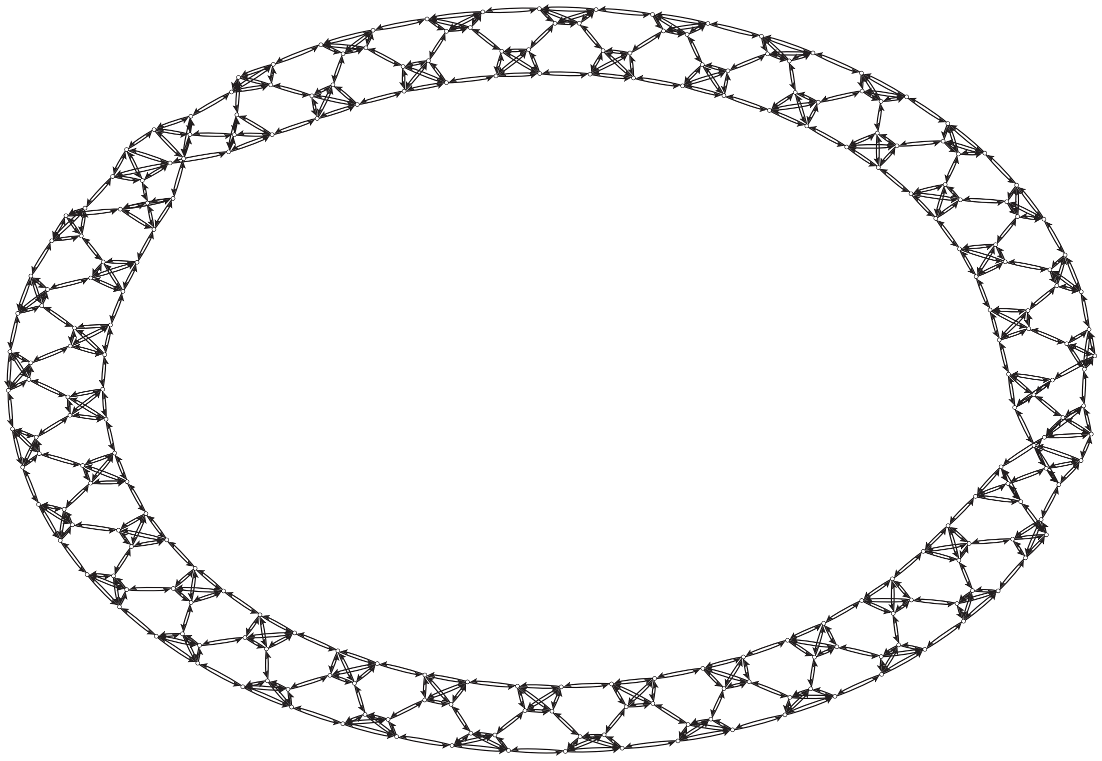

Figure S12: Structure of the lattice network constructed. *Notes:* 240 agents are grouped in cliques of four, each having exactly one tie to a non-clique member. The network data are generated with Matlab and the network structure is visualized by the authors with the software Pajek version 5.02.

11 Performance of Indirect reciprocity strategies - Lattice Network

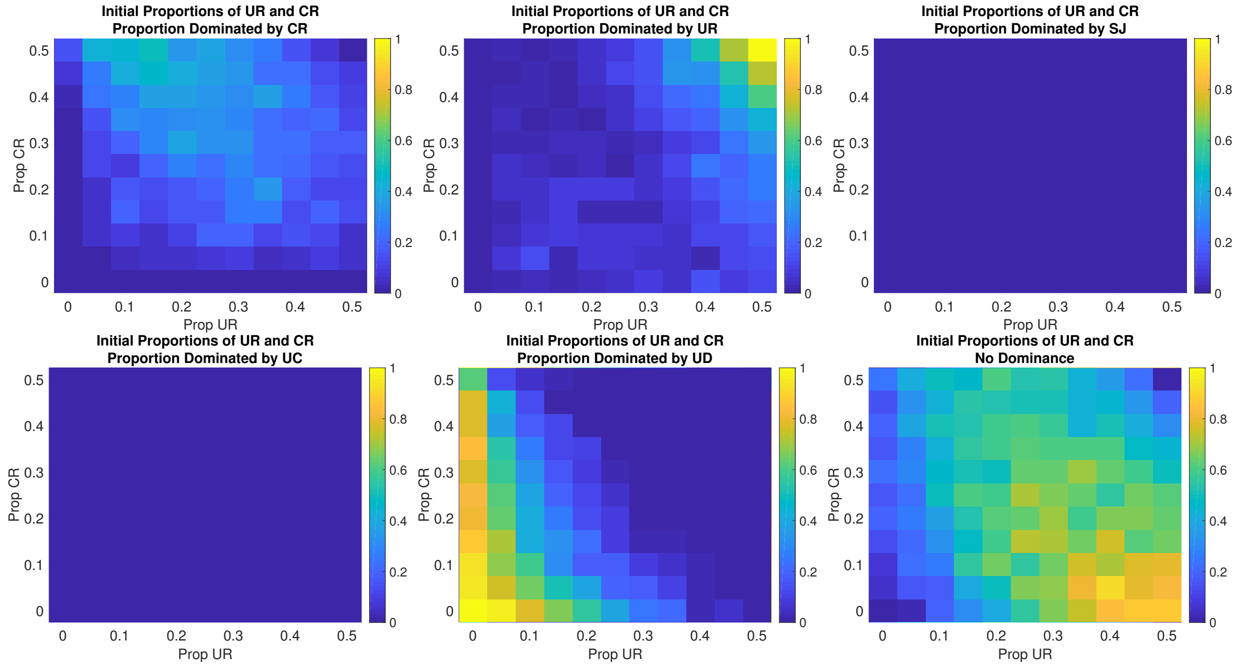

Figure S13: Effect of the Initial Proportion of UR and CR on the final proportion of simulations dominated by each strategy. *Notes:* The evolutionary update rule is copy-the-best. Domination is defined as one strategy being adopted by at least 90% of agents at the end of the simulation. Results report the proportion of simulations dominated by each strategy. For each parameter combination 100 simulations are run on a lattice networks of 240 individuals are in cliques of 4 and have exactly one ties to a non-clique member. Beside $P_{evo} = 0.05$ and $P_{for} = 0$. The initial proportion of UR is indicated on the x-axis the initial proportion of CR on the y-axis, the remaining population is initialized as equally divided among UC, UD and SJ (TFT are absent). *Top Left Panel*: Proportion of simulations dominated by CR, *Top Central Panel*: Proportion of simulations dominated by UR, *Top Right Panel*: Proportion of simulations dominated by SJ, *Bottom Left Panel*: Proportion of simulations dominated by UC, *Bottom Central Panel*: Proportion of simulations dominated by UD, *Bottom Right Panel*: Proportion of simulations with no dominating strategy.

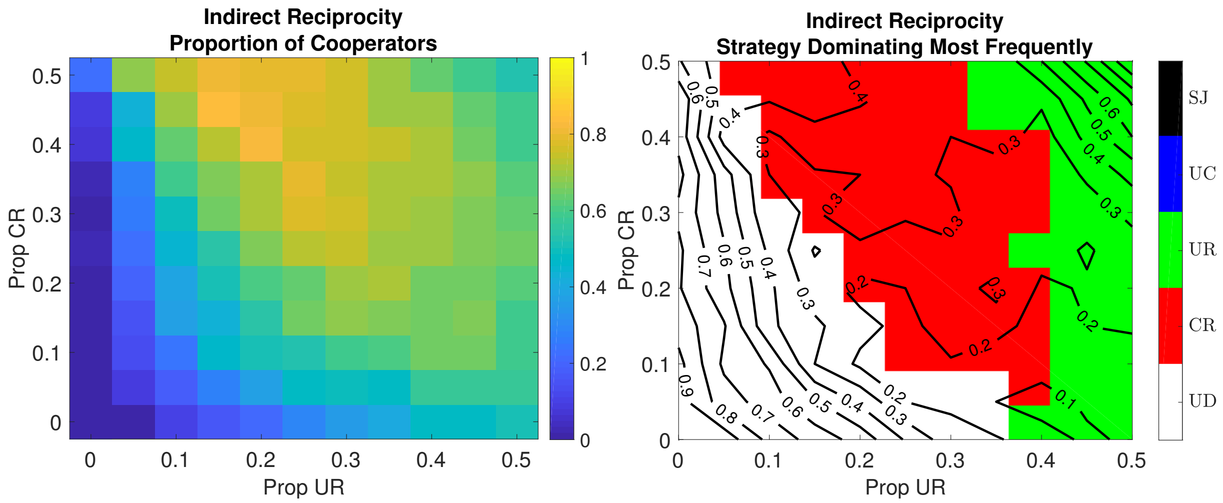

Figure S14: Effect of the Initial Proportion of UR and CR on the final proportion cooperators (*Left Panel*) on the type of agent that dominates more often in absolute terms (*Right Panel*). *Notes:* Blue corresponds to UD, yellow to CR, red to UR. 100 simulations are run on a lattice networks of 240 individuals divided in cliques of 4 and have exactly one ties to a non-clique member. $P_{evo} = 0.05$; $P_{for} = 0$; the initial proportion of UR is indicated on the x-axis the initial proportion of CR on the y-axis, the remaining population is initialized as equally divided among UC and UD (TFT is absent).

12 Convergence in actions

In the main text, simulations are considered as converged (and thus stop) when all agents become of a single strategy type. The level of cooperation registered is the one obtained at this moment. It is important to note, however, what happens in the long run to the proportion of cooperation acts when only one type of strategy is left in the population. Trivially, in a TFT-only population the long run level of cooperation is equal to the one obtained when strategies converged. TFT gets locked in homogenous choice or in a mis-coordination cycle of cooperation and defection in each dyad. More interesting are the cases of Connected Reciprocity and Unconnected Reciprocity. For UR, the proportions of cooperation either converges toward full cooperation or toward no cooperation. As one would expect, the probability of the latter event to happen is decreasing in the initial proportion of cooperation. To the contrary, for a CR-only population the level of cooperation present is preserved essentially at the same level (Figure S15).

The example reported in Figure S16 (see Supplementary Information File 2 for an animated version of this figure) shows instead that when a population becomes characterized uniquely by UR agents, it goes through cycles that result in levels of cooperation close to

the initial ones.

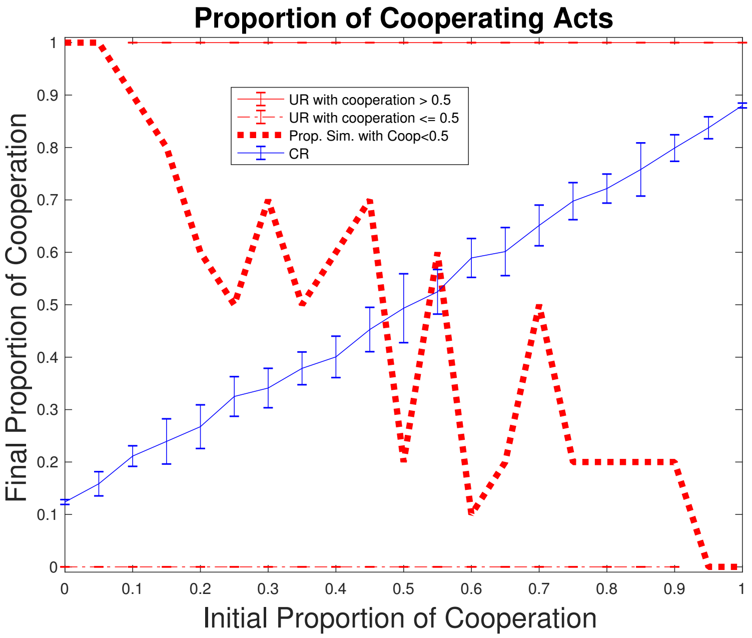

Figure S15: 100 simulations are run with populations made of only one type of agent (CR or UR). *Notes:* Averages and standard deviations of final proportions of cooperation are reported for CR populations (blue line) and UR population (in red) for different initializations of the proportion of cooperative acts. For the latter population results are reported for simulations with more (continuous red line) and less (dash-and-point red line) than 1/2 acts of cooperation. For UR strategy also the proportion of simulations with a proportion of acts of cooperations of less than 1/2 is also reported (dotted line). Simulations are run on a lattice networks of 240 individuals in cliques of 4 and have exactly one ties to a non-clique member. $P_{evo} = 0.05$; $P_{for} = 0$.

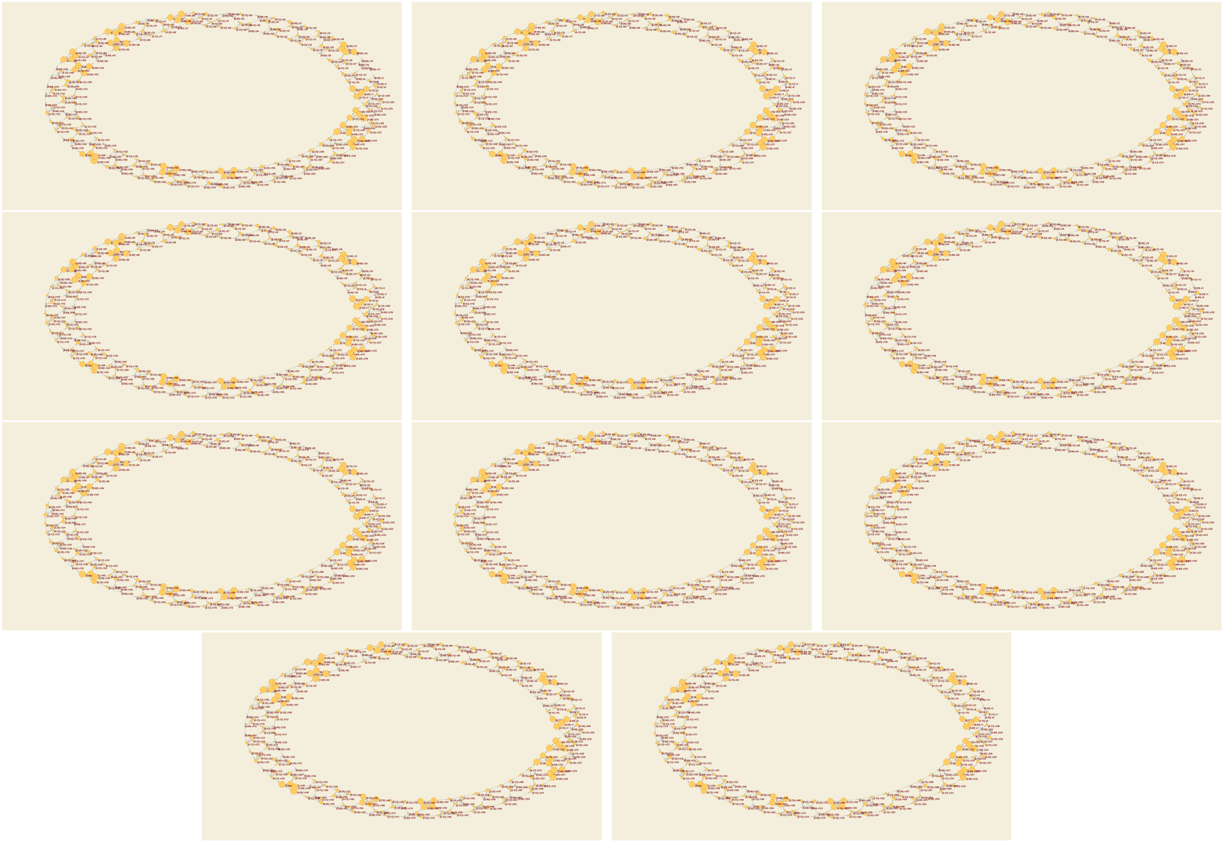

Figure S16: Example of cycle in UR only populations. *Notes:* 11 last time steps of the simulations are displayed (left to right, top to bottom). Simulations are run on a lattice networks of 240 individuals in cliques of 4 and have exactly one ties to a non-clique member.
